# Reconstruction of lower eyelid defects after the excision of basal cell carcinoma

**DOI:** 10.22336/rjo.2020.64

**Published:** 2020

**Authors:** Cătălin Gheorghe Bejinariu, Siramona Popescu, Christiana Diana Maria Dragosloveanu, Silviu Adrian Marinescu

**Affiliations:** * Department of Plastic and Reconstructive Surgery, “Bagdasar-Arseni” Clinical Emergency Hospital, Bucharest, Romania; ** Clinical Ophthalmology Emergency Hospital, Bucharest, Romania

**Keywords:** lower eyelid reconstruction, basal cell carcinoma, tumor excision, eyelid defect

## Abstract

**Objectives:** The paper presents the reconstructive options of the lower eyelid region in patients who have benefited from the excision of basal cell carcinomas.

**Methods:** The study was based on the clinical and evolutionary particularities analyzed in a series of cases, the patients benefiting from excision and reconstruction for the treatment of basal cell carcinomas located at the level of the orbital region.

**Results:** Following the surgical treatment, the local evolution was favorable with a good functional recovery. The aesthetic results were strongly influenced by the stage of the neoplasm, the final aspect being satisfactory for the patients included in the study.

**Conclusion:** Early diagnosis and rapid and effective surgical treatment are associated with favorable results from a functional and aesthetic point of view. Delayed surgery and treatment of relapses are associated with increased risks and inferior results.

## Introduction

In the last decade the incidence of basal cell carcinoma (BCC) has had an ascending trend, the specialized literature showing that approximately 70–80% of all skin carcinomas are represented by BCC [**1**]. This type of cutaneous neoplasm has a low mortality rate (< 0.1%), but its tendency to invade the neighboring anatomical structures has a strong impact on the functional and cosmetic results of the surgical treatment [**2**,**3**]. 

This paper aims to present the surgical treatment of the lower eyelid post-excisional defects, approaching both simple and large defects, or after secondary interventions for relapses. 

Generally, eyelid reconstruction must respect two principles: first and foremost, the functional principle, of protecting the eye, and secondly, the aesthetic principle [**4**]. The upper eyelid must be mobile, dynamic, in order to ease the sight and eye opening and, at the same time, to ease closing the eye and protect the cornea. On the other hand, the lower eyelid can be static [**5**]. 

The main cause of the eyelid tissue loss is represented by cutaneous tumors, especially basal cell carcinomas. For tumor excision, several guidelines should be followed:

• For superficial tumors, situated more than 5 mm from the free border of the lid, it is recommended to perform musculocutaneous excision;

• For carcinomas invading the free border of the eyelid and/ or with deep invasion, with an unaffected septum, whole plane excision is indicated;

• In case of septum invasion (with periorbital fat invasion), orbit exenteration is recommended, the excision being enlarged to the adjacent bones, if the tumor invasion demanded it.

The armamentarium of the plastic surgeon includes multiple reconstructive procedures that can be used to cover soft tissue defects and to recover the functionality of the eyelid region [**6**]. It should be noted that all these procedures are based on the guidelines presented above.

## Materials and methods – Case Reports

Partial or total destruction of the lower eyelid can easily be managed by an experienced surgeon. In case of lower eyelid reconstruction using resources from the upper eyelid, the latter should not be exposed to risks related to functionality.

Lower eyelid reconstruction is a dual plane reconstruction, corresponding to the 2 lamellas: 

• A tarsal-conjunctive plane, which is an indispensable “shield” for preventing ectropion or entropion; the reconstruction of this plan uses a mucous-cartilaginous nasal graft for the posterior lamella;

• A cutaneous plan, built from a local flap for the anterior muscular-cutaneous lamella.

• Reconstruction of the orbicular muscle and the cilia border is not necessary for the lower eyelid. 

In case of superficial tissue loss, the free lid margin has to be preserved if possible, the therapeutic approach including guided scarring, suturing, full thickness skin grafting following the aesthetic units’ principles, local flaps from either the remaining lower lid (rotational, advance or transposition flaps) or from the upper eyelid (cutaneous, muscular-cutaneous, with internal or external pedicle).

**Fig. 1:**
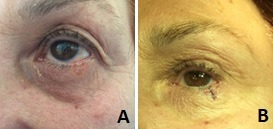
Lower eyelid reconstruction A. Preoperative aspect; B. Postoperative aspect

In case of lower eyelid reconstruction after segmented tissue loss, the Mustardé principles of the quarters are applied [**7**], so that depending on skin laxity, a defect of less than a quarter of the eyelid (or even a third of the eyelid in old patients) can be directly sutured (**Fig. 1**).

In case of defects between a quarter and a half of the lower eyelid, direct suture with or without external cantholysis can be performed in old patients; in the rest of the cases, the defect can be covered using temporo-jugal rotational flap (Mustardé), with the incision of the external commissure and of the inferior half of the external palpebral ligament (orbital septum included).

For tissue losses of more than half of the eyelid, the following solutions can be performed:

• Temporo-jugal rotational flap (Mustardé), doubled by a chondro-mucosal graft as tarsal support;

• Transpositional musculocutaneous flaps from the upper eyelid with a single (internal or external) or double pedicle, reinforced with a nasal chondro-mucosal graft, which can be harvested from alar (Texier procedure [**8**]), triangular or septal cartilages. If the defect reaches cheek area, the flap can be completed in the inferior part with a full thickness skin graft from the retro auricular region. 

For subtotal or total tissue defects, eyelid reconstruction is performed using temporo-jugal rotational flaps (Mustardé), transposition flaps, such as Texier flaps, orbital, nasal and cheek flaps with chondro-mucous graft, frontal flaps with septal cartilage graft, temporal flaps with inferior pedicle or external frontal flaps (rarely used).

## Results

**Fig. 2:**
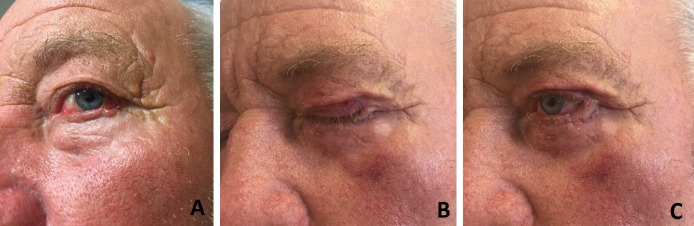
Lower eyelid reconstruction using upper eyelid muscular-cutaneous flap with external pedicle and tarsal ligament suspension to periosteum or external orbital process – A. Preoperative aspect; B. Postoperative aspect - complete occlusion; C. Postoperative aspect - complete opening

From the series of cases, four cases were selected in order to present the results of the previously mentioned procedures:

**Case 1** - Post-excisional defect coverage after basal cell carcinoma using upper eyelid muscular-cutaneous flap with external pedicle and tarsal ligament suspension to periosteum or external orbital process (**Fig. 2**).

**Fig. 3:**
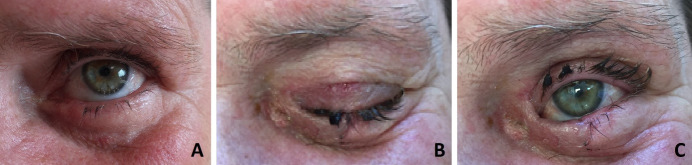
Lower eyelid reconstruction using upper eyelid muscular-cutaneous flap with internal pedicle and lacrimal duct reconstruction using tubular silicone prosthesis for duct patency – A. Preoperative aspect; B. Postoperative aspect - complete occlusion; C. Postoperative aspect - minimal damage to the eyelid function.

**Case 2** - Post-excisional defect coverage after basal cell carcinoma using upper eyelid muscular-cutaneous flap with internal pedicle and lacrimal duct reconstruction using tubular silicone prosthesis for duct patency (**Fig. 3**).

**Fig. 4:**
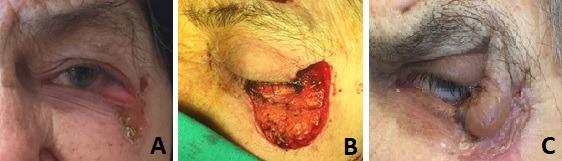
Lower eyelid reconstruction using upper eyelid muscular-cutaneous flap with external pedicle and chondro-mucous nasal graft and full thickness retro-auricular skin graft placed inferior to the lower lid - A. Preoperative aspect; B. Intraoperative aspect - complete excision; C. Postoperative aspect - moderate impairment of the eyelid function with temporary oedema.

**Case 3** - Post-excisional defect coverage after basal cell carcinoma (note the advanced palpebral retraction made by the tumor) using upper eyelid muscular-cutaneous flap with external pedicle and chondro-mucous nasal graft and full thickness retro-auricular skin graft placed inferior to the lower lid (**Fig. 4**). 

**Fig. 5:**
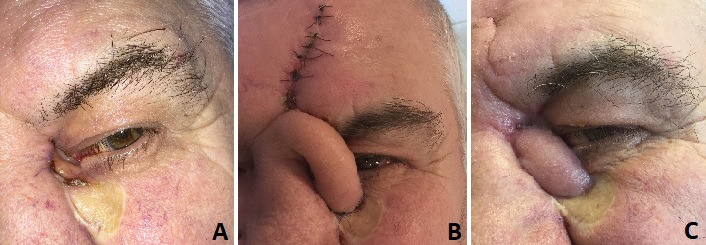
Lower eyelid reconstruction - A. Preoperative aspect; B. Postoperative aspect (time 1) - covering the post excisional soft tissue defect with a frontal flap; C. Postoperative aspect (time II) - pedicle section of the frontal flap - moderate impairment of the eyelid function

**Case 4** - Soft tissue coverage in the internal angle of the eye, including the lower lid, using fasciocutaneous frontal flap after basal cell carcinoma relapse. The first step of the surgical treatment included tumor excision and tissue defect coverage with inferior pedicle frontal flap. The second step involved pedicle sectioning and rearrangement of the flap segments (**Fig. 5**). 

## Discussions

The increased incidence of basal cell carcinoma located in the facial region, the lethal potential and the mutilating nature of this disease are fundamental elements that have aroused the interest of the international scientific community dedicated to the diagnosis and treatment of this type of disease. International statistics place basal cell carcinoma in the first place in the classification of the most common neoplasms, ultraviolet radiation, immunosuppression, and the Caucasian race being the main risk factors for the occurrence of this type of neoplasm [**9**].

Regarding the therapeutic protocol, the complete excision of the tumor is the fundamental element that leads to good results that are stable over time. Recurrences of basal cell carcinoma are often associated with a challenging evolution, marked by postoperative complications and relapses. The specialized literature shows that the risk of recurrence related to basal cell carcinoma located on the face is strongly influenced by incomplete excision, the infiltrative and micronodular type, as well as the existence of a history of surgical interventions involving excision followed by recurrence [**10**,**11**].

Planning the orbital-palpebral surgery involves certain measures, such as avoiding contact between the disinfecting agent and the eye, in order to prevent keratitis and corneal erosion and protecting the cornea using artificial tear drops, saline serum or methyl-cellulose, while avoiding long compression of the eye ball [**12**,**13**]. Other important aspects regarding palpebral reconstruction are inspecting the pupil, considering that an irregular mydriasis may suggest an intra-orbital diffusion of vasoconstrictors, using only dressings soaked in saline serum and ensuring a rigorous hemostasis [**14**]. It is mandatory not to inject vasoconstrictor agent posterior to the septum in order to prevent vascular incidents. The surgical node is not to be placed in direct contact with the cornea - source of mechanic laceration. Other indications are to rinse the cornea thoroughly with saline serum and inspect it at the end of the surgery (the cornea should be clear and hydrated) and to ensure that the occlusive dressing of the eye is not obstructive and topical antibiotics in the inferior conjunctivae sac can be a good option in selective cases in order to avoid cornea dryness. 

Nevertheless, the eyelid region requires surgical finesse and challenges even for the most experienced surgeons. Neglected cases often require the use of complex combinations of reconstructive procedures. Establishing the compatibility of different techniques is the main element that constitutes the foundation for solving complex cases and can be considered as a true link between specialties, the involvement of the ophthalmologist being essential in this stage of the reconstructive protocol.

## Conclusions

Basal cell carcinomas manifest their malignancy through their local invasion characteristics that produce local damages. When situated near sensory organs (such as the eye), basal cell carcinomas have a huge impact on their functionality affecting the sensory abilities. The role of the plastic surgeon is to first reestablish the functionality of the invaded organ after radically excising the tumor. Following, the aesthetic aspect has to be considered in order to improve quality of life. Considering all these principles, the standard of care is represented by reconstruction with local flaps and composite grafts (chondro-mucous cartilage) and full thickness skin grafts with similar characteristics of the recipient (color, thickness). Compared to skin grafts, local flaps offer the advantage of higher quality tissues, therefore being a much more effective reconstructive option. On the other hand, there are some aesthetic disadvantages, such as pathological scarring, delayed healing of the donor or recipient sites, prolonged oedema after surgery, graft necrosis and flap retraction. In the vast majority of the situations, a multidisciplinary approach involving the plastic surgeon and ophthalmologist is beneficial in finding an optimal and personalized solution for the patient. 

**Conflict of Interest**

The authors state no conflict of interest.

All authors agree with the publication of this manuscript.

**Informed Consent**

The patient was informed and signed the informed consent to participate in this study.

**Authorization for the use of human subjects**

This clinical investigation complies with the standards of the Ethics Committee of the “Bagdasar-Arseni” Clinical Emergency Hospital and adheres to the principles of the Declaration of Helsinki.

**Acknowledgements**

None.

**Sources of Funding**

None.

**Disclosure**s

None.

## References

[1] Al-Qarqaz F, Marji M, Bodoor K, Almomani R, Al Gargaz W, Alshiyab D, Muhaidat J, Alqudah M (2018). Clinical and Demographic Features of Basal Cell Carcinoma in North Jordan. J Skin Cancer.

[2] Ciążyńska M, Narbutt J, Woźniacka A, Lesiak A (2018). Trends in basal cell carcinoma incidence rates: a 16-year retrospective study of a population in central Poland. Postepy Dermatol Alergol.

[3] Goldenberg G, Karagiannis T, Blanche Palmer J, Lotya J, O'Neill C, Kisa R, Herrera V, Siegel DM (2016). Incidence and prevalence of basal cell carcinoma (BCC) and locally advanced BCC (LABCC) in a large commercially insured population in the United States: A retrospective cohort study. Journal of the American Academy of Dermatology.

[4] Bejinariu CG, Dragosloveanu CDM, Marinescu SA (2020). Complex reconstruction of the orbitofrontal regions using three regional flaps after orbital exenteration for the treatment of basal cell carcinoma. Romanian Journal of Ophthalmology.

[5] Codner MA, Weinfeld AB (2007). Pr47 comprehensive eyelid reconstruction. ANZ J Surg.

[6] Mathijssen IM, van der Meulen JC (2009). Guidelines for reconstruction of the eyelids and canthal regions. J Plast Reconstr Aesthet Surg.

[7] Mustardé JC (1969). Repair and Reconstruction in the orbital region. A practical guide.

[8] Texier M, Preaux J (1981). Reconstruction de la paupiere inferieure par greffe chondro-muqueuse alaire et lambeau cutaneo-musculaire palpebral superieur. Ann Chir Plast.

[9] Young SM, Amrith S, Wu B, Nga ME, Sundar G (2019). Basal Cell Carcinoma. In: Amrith S, Sundar G, Young S. (eds) Ocular Adnexal Lesions.

[10] Armstrong LTD, Magnusson MR, Guppy MBP (2017). Risk factors for recurrence of facial basal cell carcinoma after surgical excision: A follow-up analysis. Journal of Plastic, Reconstructive & Aesthetic Surgery.

[11] Striker M, Gola R (1990). Chirurgie plastique et reparatrice des paupieres et de leurs annexes.

[12] Bejinariu CG, Marinescu S (2019). Lower Limb Salvage Using the Medial Hemisoleus Flap Associated with the Reverse Sural Flap. J Med Life.

[13] Moesen I, Paridaens D (2007). A technique for the reconstruction of lower eyelid marginal defects. Br J Ophthalmol.

[14] Revol M (2012). Manuel de chirurgie plastique, reconstructice et esthetique.

